# A Systematic Review of the Recent Techniques Commonly Used in the Diagnosis of *Mycoplasma bovis* in Dairy Cattle

**DOI:** 10.3390/pathogens12091178

**Published:** 2023-09-19

**Authors:** Hedmon Okella, Karen Tonooka, Emmanuel Okello

**Affiliations:** 1 Veterinary Medicine Teaching and Research Center, School of Veterinary Medicine, University of California Davis, Tulare, CA 93274, USA; 2 Department of Population Health and Reproduction, School of Veterinary Medicine, University of California Davis, Davis, CA 95616, USA

**Keywords:** detection, mycoplasma, bovine mastitis, methods

## Abstract

Early detection of Mycoplasmal mastitis is greatly hampered by late seroconversion, slow growth of Mycoplasma organisms, intermittent shedding, and the high cost of diagnostic tests. To improve future diagnostic development, examining the available techniques is necessary. Accordingly, the present study systematically reviewed *M. bovis* diagnostic studies published between January 2000 and April 2023 utilizing the Preferred Reporting Items for Systematic Reviews and Meta-Analysis (PRISMA) protocol. The protocol registration was performed according to the Open Science Framework (osf.io/ug79h), and the electronic search was conducted in the World Catalog, Mendeley, ProQuest, ScienceDirect, Semantic Scholar, PubMed, Google Scholar, Prime Scholar, and PubMed Central databases using a Boolean operator and inclusion and exclusion criteria. Of the 1194 pieces of literature retrieved, 67 studies were included. Four broad categories of up to 16 diagnostic approaches were reported: microbial culture, serological, DNA-based, and mass spectrometry. Overall, DNA-based techniques were the most published (48.0%), with recombinase polymerase amplification (RPA) and loop-mediated isothermal amplification (LAMP) as the most promising user-friendly, equipment-free techniques. On the other hand, mass spectrometry was reported as the least utilized (2.9%) given the high equipment cost. Though costly and laboratory-allied, DNA-based techniques, particularly PCRs, were reported as the most rapid and specific approach.

## 1. Introduction

Bovine mastitis, an infection of udder tissue due to bacterial infection, is an increasingly prevalent disease in dairies [1]. The disease is responsible for substantial economic losses for producers each year [2]. Of the four most common Bovine mastitis-causing pathogens, viz. *Mycoplasma bovis*, *Mycoplasma canadense*, *Mycoplasma californicum*, and *Mycoplasma bovigenitalium* [3], *M. bovis* is the most common pathogenic [4] and highly contagious [5]. Besides bovine mastitis, *M. bovis* is responsible for otitis [6], genital disorders and abortion [7], bovine respiratory disease (BRD) [8], keratoconjunctivitis [9], and chronic pneumonia and poly-arthritis syndrome (CPPS) [10].

*Mycoplasma bovis* are relatively small with a genome size of ~953 kbp [11] and a low GC ratio and lack of a cell wall [12,13]. Because of their small genome, Mycoplasma cannot perform critical metabolic pathways, thus leading to a parasitic and demanding lifestyle.. Their lack of a cell wall makes them resistant to β-lactams and other antimicrobial compounds, thereby limiting the effectiveness of antibiotic treatments [14]. *M. bovis* infection spreads quickly, primarily through animal-to-animal contact. Still, it can also spread through contact with personnel or equipment, airborne transmission, and during artificial insemination, rendering them the most contagious mastitis-causing *Mycoplasma* spp. [15,16]. 

Mycoplasma mastitis is considered untreatable with substantial negative impacts on milk production [17] and overall cow weight gain [18], which amounts to severe economic losses to producers. Losses due to mastitis caused by *M. bovis* in the United States were estimated at USD 108 million per year with herd infection rates of up to 70% [19,20]. In 2014, an estimated 99.7% of the dairies in the United States had mastitis, and the average herd prevalence of clinical mastitis was 24.3% [21]. In California alone, *M. bovis* was associated with up to 52.2% of Mycoplasma mastitis [22]. 

Considering that *M. bovis* mastitis lacks effective treatment, control of the infection in a herd solely relies on early identification, isolation, and culling of infected animals. Unfortunately, the clinical and pathological signs of *M. bovis*-infected cattle are non-specific [23], thereby complicating its diagnosis. Thus, rapid, sensitive, and accurate animal testing is needed to control potential outbreaks. To date, microbial cultures, polymerase chain reactions (PCRs), and serological diagnosis using an ELISA remain the principal techniques employed in *M. bovis* detection [24]. However, the economic losses caused by *M. bov* is diagnosis and the need for user-friendly, field-applicable diagnostics requires an extensive review of the available techniques. Previous reviews on *M. bovis* diagnosis have focused different aspects including transmission and detection [25], control [17], mastitis importance [26], epidemiology [27], occurrence and control [23], diagnosis and control [28,29,30,31], and virulence and the host immune response in infected cattle [30]. Such reviews should have systematically processed the most recent *M. bovis* diagnostic techniques in much detail. Thus, this study systematically examined recent diagnostic techniques for *M. bovis*, including their benefits, accuracy, and limitations, to aid in the development of future diagnostics.

## 2. Materials and Methods

### 2.1. Literature Search Strategies 

The present systematic review was executed in compliance with the Preferred Reporting Items for Systematic Reviews and Meta-Analysis (PRISMA) [32] protocols registered in Open Science Framework (osf.io/ug79h). Here, the Boolean operator [(diagnos*) or (detect*) AND (“bovine mastitis”) or (“mycoplasma bovis mastitis”) or (“bovine mycoplasmosis”)] was used to carefully search World Catalog, Mendeley, ProQuest, ScienceDirect, Semantic Scholar, PubMed, Google Scholar, Prime Scholar, and PubMed Central databases for literature published in English between January 2000 and April 2023. 

### 2.2. Eligibility Criteria

To ensure that only relevant studies were included, the articles were first checked for any duplicates using their titles. The abstracts of potential articles were then screened to determine their eligibility. Only original studies that reported on the diagnosis of *M. bovis* and that met strict inclusion criteria were included. Review articles, both published and unpublished, were not considered. Articles related to other types of mastitis and those that were not available in English were also excluded. Finally, articles that did not provide information on *M. bovis* detection or whose full text was not available at the time of the search were excluded.

### 2.3. Data Extraction and Appraisal

A multiple-assessment approach across authors was used to identify, screen, and select studies based on PRISMA guidelines [32]. During the research process, regular discussions were held among the team while iterating through screening, analysis, and synthesis. To evaluate the literature, the Guide Evaluation of Qualitative Research Studies (GEQRS) was utilized [33]. The assessment also utilized Joanna Briggs Institute’s critical appraisal checklist for studies reporting on prevalence data fields [34]. Data were then extracted using the guidance for conducting systematic scoping reviews [35,36]. To check for the consistency and reliability of studies, two authors conducted the quality appraisal approach [32,37]. Articles that met less than 70% of the assessment criteria signified high bias or risk and were dropped [37]. Table 1 presents a summary of the articles that were included, showing the type of biological sample, diagnostic methods used, the detection unit, percentage of *M. bovis* detected, as well as the country of origin, authors, and year of the study.

## 3. Results and Discussions 

**Figure 1:**
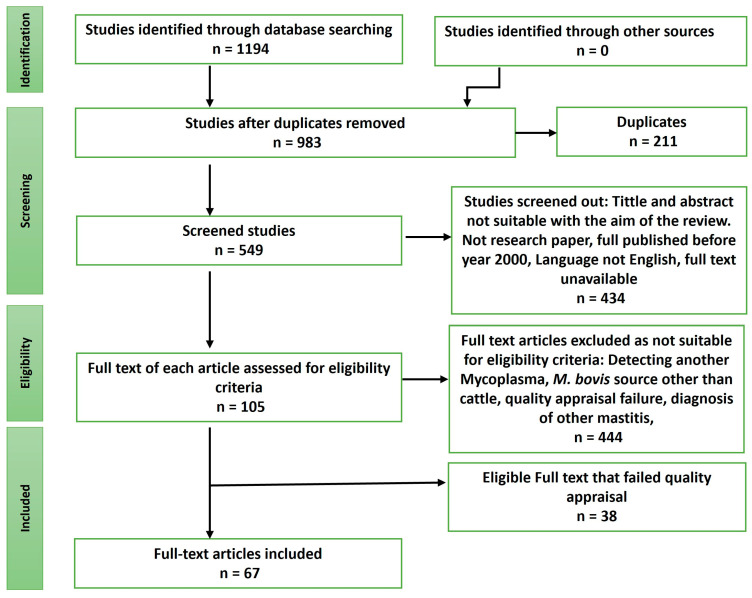
PRISMA flow diagram on recent techniques for *M. bovis* detection. The 1194 identified documents were screened and appraised, from which 67 of them were included in this study. All studies were accessed through an electronic database search.

A total of 1194 articles were obtained through the database searches. After the removal of duplicates and assessing the full-text articles based on the inclusion and exclusion criteria, only 67 primary studies were qualified (Figure 1). The highest proportion of these studies were conducted in the United States (11.9%), followed by Australia (8.9%), and India (7.4%). Most of the studies included in the analysis utilized milk samples for the diagnosis of *M. bovis* (41.8%), while a few used sera (17.9%), and the least used sample type was serum (1.5%). 

**Figure 2:**
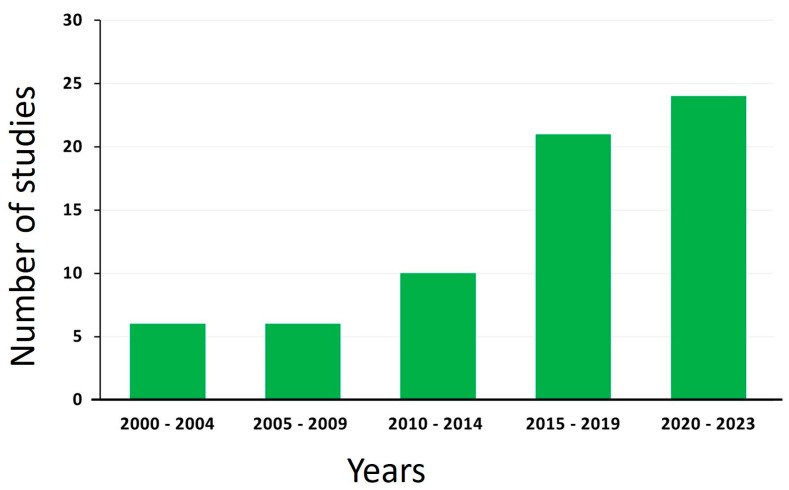
The number of studies by years. The number of studies on detection of *M. bovis* increased since 2000 with the highest number (24) recorded between January 2020 and April 2023. The least number of studies (06) were between 2000–2004.

To accurately diagnose *M. bovis* mastitis and differentiate it from other types of mastitis, laboratory testing, such as bacterial culture and identification, or molecular tests, such as a PCR, is required. Unfortunately, the expense of molecular tests makes them impractical for routine use. Additionally, the lengthy process for bacterial identification and turnaround time makes it less effective for guiding management decisions. The diagnosis process is further complicated by intermittent bacterial shedding and subclinical infection states. The present study evaluated over 14 diagnostic methods broadly categorized into bacterial culture, serology, DNA-based techniques, and mass spectrometry. Most of the studies included in the review (24 out of 67) were conducted between 2020–2023, followed by those reported between 2015–2020 (21 of the 67). The least number of studies were encountered in the early 2000s (6 out of 67) (Figure 2). 

Overall, DNA-based techniques were the most reported technique (48.0%) for the diagnosis of bovine mastitis, followed by serology (26.5%), while mass spectrometry was the least utilized technique (2.9%). 

### 3.1. Microbial Culture 

In this study, although culturing was once considered the gold standard for *M. bovis* detection, it was the third most reported technique, likely due to the evolution of new techniques [23]. In addition, the culture method is comparatively inexpensive [5] with a contemporary cost range of USD 5 to USD 6.5 depending on the laboratory used [82]. According to a previous study, the culture method successfully detected 98% of *M. bovis-infected* caseo-necortic lung tissues in Canadian feedlot beef calves [93]. Similarly, studies by Sickles et al., 2000 and Wilson et al., 2009 reported the successful detection of *M. bovis* in 13.7% and 3.2% bulk tank milk samples, respectively [90,100]. Since the culture method can detect the viable *Mycoplasma* species with a high specificity and sensitivity (101–102 CFU/mL), the technique remains vital for the laboratory diagnosis of *M. bovis* [29,102]. Moreover, the centrifugation of milk samples and plating the resuspended pellets instead of direct sample plating further improves the detection of *Mycoplasma* spp. by four-fold [103].

A major limitation of the culture method is that it can only identify Mycoplasma organisms to the genus level [104]. Moreover, the culture method is a laborious and time-consuming process, as growth is only typically observed after three days with a characteristic “fried-egg” appearance [105]. Negative plates should be reexamined after 7 days of incubation [104,106]. Unfortunately, due to the highly contagious nature of *M. bovis* and the long culture period, it is challenging to implement timely responses in cows with positive culture results. This delay can lead to the spread of the disease to other animals within the herd. *M. bovis* has very specific growth requirements and is a slow-growing organism that can easily be overgrown by contaminating bacteria. Growth contamination can, however, be avoided by supplementing the media with antibiotics. The principal media commonly used for the detection of *M. bovis* infections include Eaton’s [106], Hayflick’s [107], and modified PPLO [108]. These media require good laboratory settings, such as a carbon dioxide incubator, as well as a skilled workforce for the successful isolation of *M. bovis*. 

Ordinarily, the detection of *M. bovis* infection in a culture requires the cattle to be shedding viable organisms. Even so, studies have shown that there is intermittent shedding in chronic and subclinical mycoplasma mastitis cases, with up to 56 days without shedding in cattle with chronic mastitis [27,29,109]. This calls for alternative techniques like serology that do not rely on the shedding of viable organisms by the cattle. Additionally, culture methods are unable to differentiate between closely related species. *Mycoplasma* species cannot be distinguished from *Acholeplasma*, a species considered non-pathogenic, on a modified Hayflick medium without additional tests. Essentially, aide tests, such as digitonin or nisin disk diffusion assays, or PCRs are inevitable [110]. Overall, microbial culture techniques have limitations that affect their effectiveness and efficiency in diagnosis [111]. These limitations include time, field applicability, and accuracy.

### 3.2. Serology 

Serological techniques can detect specific antibodies against *M. bovis* in milk, plasma, or serum samples within two weeks of infection. Since only the antibody response is detected, it is not necessary for the cows to be actively shedding the pathogens at the time of sample collection, unlike with the culture method [18], and the antibody level remains high for several months [112]. This particular attribute renders serology a reliable technique in herds where heavy antibiotics use and chronic infections usually impede *M. bovis* isolation [106]. However, serology, just like the culture technique, is laboratory-based and demands trained staff. During this review process, five serological techniques were encountered, and these include, an enzyme-linked immunosorbent assay (ELISA) [43,78], immunohistochemistry (IHC) [113], the direct fluorescent antibody technique (DFAT) [114], an immunobinding test (IBT) [115], sodium dodecyl-sulfate polyacrylamide gel electrophoresis (SDS-PAGE,) and immunoblotting [83]. 

#### 3.2.1. Enzyme-Linked Immunosorbent Assay 

This is the most used serological technique for detecting *M. bovis-specific* antibodies. Most of the published serological studies (70.4%) employed an ELISA in diagnosing *M. bovis*. The ELISA is mostly utilized in herd-level diagnosis rather than individual animal testing, with results available within two days [27,116]. Through the ELISA technique, a large number of samples can be screened; thus, it is ideal for surveillance or biosecurity programs [98]. To date, various commercial ELISA kits are available, including *Bio X*, *CHECK-IT M. bovis-Sero*, *IDEXX*, *Biovet*, and *MilA*, among others, with the Bio-X *M. bovis* ELISA kit being the most utilized kit. A study conducted in Turkey [80] showed that the BIO-X *M. bovis* antibody ELISA kit detected up to 35.4% of the *M. bovis*-specific antibodies in 127 tracheal swab samples across seven geographically distinct farms in Turkey. Even higher percentages of *M. bovis-*specific antibodies (38% and 46%) were reported using the same kit in Nigeria [78] and Australia [70]. 

As much as the ELISA technique is generally less labor-intensive and time-consuming than culture methods, seroconversion may take 2–3 weeks before antibodies can be detected and may lead to false negative results [117]. In addition, uncertainty due to cross-reactivity with other closely related organisms decreases ELISA specificity [118] and hence the need for complementary techniques for an accurate diagnosis. Additionally, the presence of anti-*M. bovis* antibodies does not mean the animal has or is shedding viable pathogens. Still, the presence of antibodies could indicate infection at a site other than the mammary gland or could be a result of maternal or natural antibodies [17,27]. It is therefore recommended that the results of the ELISA be used in addition to DNA-based techniques for bulk-tank milk testing. While bulk-tank sampling may achieve fewer false positives, it decreases the sensitivity of the test to about 43.5%, making it difficult to interpret the results and inform appropriate action for individuals. However, it can still be useful for monitoring herd health [119]. 

#### 3.2.2. SDS-PAGE and Western Blotting

The antigenic structure of mycoplasma strains can be compared using SDS-PAGE and western blotting. This technique can also reveal the host animal’s humoral immune response patterns [120]. The technique was first utilized to identify 34 isolates of *M. bovis* with a high degree of similarity [102]. Later, potential *M. bovis* antigenic proteins were identified after separating them on sodium dodecyl-sulfate polyacrylamide gel electrophoresis [84]. The same technique was used to distinguish *M. bovis* from *M. agalactiae* based on their native protein patterns on the gel [83]. Their study unveiled seven major immunogenic cross-reactive proteins and two important non-cross-reacting species-specific polypeptides (25.50 and 24.54 kDa) in *M. agalactiae* and *M. bovis*, respectively. This reaffirmed earlier reports that western blotting might address the cross-reactivity drawbacks of techniques like ELISAs [121].

#### 3.2.3. Immunobinding Test

The immunobinding test is one of the oldest techniques used to diagnose *M. bovis*. The technique was first used by Infante Martinez et al. (1990) to detect Mycoplasma species in milk [115]. They found the assay highly specific when monoclonal antibodies were incorporated. Later, the technique was improved to detect *M. bovis* in 42.3% (55/130) of naturally infected milk within just 110 min at the highest level of sensitivity and specificity [97]. Since then, the technique has evolved into nitrocellulose paper with monoclonal antibodies. This technique was used to detect *M. bovis* cultural isolates and swabs from the genitals of artificially infected heifers utilizing the PCR as the gold standard [122].

#### 3.2.4. Direct Fluorescent Antibody Technique 

In this technique, fluorescently labeled monoclonal antibodies are made to bind and illuminate the target M. bovis antigens directly [115,123]. A study on 120 mammary samples from slaughterhouses in Esrzurum Province, Turkey, Altun and Ozdemir (2019), detected 23.3% of M. bovis in the cattle mammary tissue using the direct fluorescent antibody technique. Similarly, other Mycoplasma species, such as *M. californicum*, *M. bovigenitalium*, *M. canadense*, *M. arginini*, and *M. alkalescens*, in milk samples from California dairies were effectively speciated at a low cost using this technique [82]. Thus, the technique remains the most preferred cost-effective and specific approach to detect and speciate Mycoplasma spp. However, generating fluorescent antibodies is a laborious and skill-demanding process. 

#### 3.2.5. Immunohistochemistry

In immunohistochemistry, *M. bovis* antigens can be detected in situ using immunochemical analysis after fixing the tissue in formalin and embedding it in paraffin [93,113]. A study on 35 commercial dairy herds in southern Brazil detected up to 91.4% of *M. bovis* in pulmonary sections of Holstein cows using immunochemistry [58]. Another study detected 73.9% of *M. bovis* antigens in 23 calves across southern Spain using the same technique. However, a lower value (18.2%) was reported from the lung tissue of 87 bovine carcasses from Indian farms showing visible pneumonic lesions at necropsy [64]. Although this serological technique is considered in situ and hence worthwhile over the culture technique, it is still labor-intensive and costly to generate fluorescent antibodies.

### 3.3. DNA-Based Techniques

**Figure 3:**
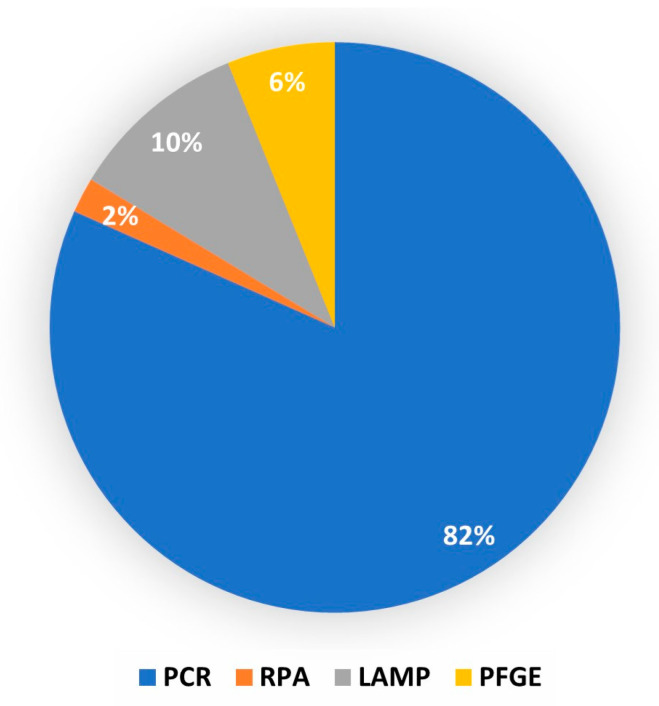
Distribution of DNA-based techniques encountered in the diagnosis of bovine mycoplasmosis. RPA, Recombinase Polymerase Amplification; PCR, Polymerase Chain Reaction; LAMP, Loop-mediated Isothermal Amplification; PFGE, Pulse Field Gel Electrophoresis. PCR technique was the the most utilized DNA-based technique (82.0%), followed by LAMP (10.0%).

*M. bovis* detection is laborious, time-consuming, and difficult to perform. Moreover, the detection of *M. bovis* through serological methods is challenging due to potential cross-reactivity. Therefore, DNA-based techniques have become the favored diagnostic approach. DNA-based techniques, particularly the polymerase chain reaction (PCR) allows for the rapid and specific detection of *M. bovis* [124]. The PCR was the most used diagnostic technique (81.6%) in this investigation, likely due to these properties (Figure 3). The PCR technique offers faster diagnosis compared to culture and serological methods, with results available in just a few hours [102], thus allowing for prompt action, such as the removal of infected cows from the herd. Further, PCR methods can specifically amplify *M. bovis* DNA, enhancing the identification of Mycoplasma species [125]. Moreover, more than one species of *Mycoplasma* as well as non-cultivable or unknown species can be detected when conventional PCR products are run through denaturing gradient gel electrophoresis [126], a property comparable to microarrays [127]. The PCR-DGGE method effectively detected 9.3% *M. bovis uvrC* genes in 713 nonpharyngeal swabs of Polish cattle [24], thus rendering it an effective and accurate technique. 

One drawback of the PCR is that non-viable bacteria can still be detected [128]. Another drawback associated with the PCR is the high cost involved. Nonetheless, sample pooling has been suggested as a cost-saving approach [57,59,82]. Over time, the methods used to diagnose *M. bovis* with a PCR have evolved. These advancements include the conventional singleplex PCR and multiplex, nested, and real-time PCRs, but they often come with additional costs. 

#### 3.3.1. Conventional PCR

The conventional PCR involves in vitro amplification of unique specific DNA targets using sequence-specific oligonucleotide primers and heat-stable polymerase enzymes [129]. Several *M. bovis* DNA targets have been reported, and they include *uvrC*, *16S rRNA*, *gyrB*, *polC*, *16S-23S rRNA ITS*, *oppD*, *vspB*, and gltX, among others. In the present study, *uvrC* (22.2%) was the most reported gene, followed by *oppD* (1.7%) and *16S-23S rRNA ITS* gene (1.4%). Moreover, other *M. bovis* genes were reported in studies that successfully diagnosed bovine mastitis using a conventional singleplex PCR [39,48,64,75,79,85,90].

Additionally, a conventional PCR variant (the multiplex PCR) in which more than a pair of primers is utilized for simultaneous amplification of multiple target sequences in a single reaction tube [130,131] was reported. Still, *uvrC* genes were the most preferred target given that *16S rRNA* genes display a low level of variation to differentiate between closely related *Mycoplasma* species, like *M. bovis* and *M. agalactiae* [132].The *multiplex* PCR was employed in the detection of *M. bovis* genes as well as in distinguishing it from *M. agalactiae* isolated from milk and nasal swabs based on the multiplex PCR amplified products [95]. In a study from Argentina, Neder and colleagues (2021) used two sets of primers (MBOUVRC2-L and MBOUVRC2-R) and could detect 7.9% (n = 38) of *M. bovis* genes in the bulk tank milk samples amidst other *Mycoplasma* species, like *M. californicum*, *M. canadense*, and *M. leachii*. 

To further improve both the sensitivity and specificity of the conventional PCR, the nested PCR evolved [133]. Unlike the multiplex PCR, a nested PCR utilizes two pairs of amplification primers in two successive rounds of a PCR [26,133,134]. In their study on field milk samples from Iowa farms, USA, Pinnow et al. (2001) detected 49.1% *M. bovis* genes in 53 milk samples using the nested PCR to a sensitivity of 5.1 CFU/mL. Later, a semi-nested PCR was reported to have detected *M. bovis* genes in milk (27.5%), nasal (30.0%), conjunctiva (12.5%), and vaginal (2.5%) samples during their first test on 40 milking Friesian–Holstein dairy cattle in Australia [96].

#### 3.3.2. Real-Time PCR 

The real-time PCR quantifies the amplified PCR products based on different fluorogenic DNA probes [135]. It has extra benefits over the conventional PCR as a faster and more sensitive alternative with no need for post-reaction handling. The sensitivity and specificity of the real-time PCR for mastitis detection may reach 100% [136,137]. However, these advantages come at a much higher financial expense, as qPCR testing for three pathogens can cost between USD 19–50 compared to a culture cost of USD 5–6.50 [82]. Should a dairy wish to run samples in-house (an on-farm PCR) to cut costs, it would still require access to specialized equipment and trained personnel, which are equally costly. In addition, an on-farm PCR is prone to high contamination that can cause false positives or a high threshold for the detection limit that can increase the possibility of false negatives. 

Several commercial qPCR kits are currently available for the detection of *M. bovis*. *PathoProof* Mastitis Major-3 by Thermo Fisher Scientific is one of the commercially developed qPCR kits capable of detecting *M. bovis* alongside two other contagious mastitis pathogens (*S. aureus* and *S. agalactiae*). The *bacto-type* Mastitis HP3 PCR kit from *QIAGEN* is another highly sensitive and specific test for the identification and differentiation of DNA from three major mastitis-causing pathogens (*M. bovis*, *S. agalactiae*, and *S. aureus)* in milk samples (quarter milk samples, pool, or bulk milk). The *Pneumo4B* and *Pneumo4V* qPCR was developed in 2020 for the detection of *M. bovis* in the tracheal aspirate samples from calves [138]. Other commercial kits include the *VetMAX™ MastiType Myco8* Kit (ThermoFischer, Warrington, United Kingdom) and *Mastit4* (DNA Diagnostics, Risskov, Denmark).

Using a real-time PCR, several studies reported the successful and accurate diagnosis of *M. bovis* mastitis worldwide. In India, Behera et al. (2018) utilized a SYBR green dye-based real-time PCR assay targeting the *uvrC* gene for the diagnosis of *M. bovis* in milk and lung tissue. Recently, targeting *polC* genes, Becker et al. (2020) reported 51.0% of *M. bovis polC* genes as positive samples in 251 nasal swabs of calves in western France. The same gene was utilized in a real-time PCR that detected 58% of *M. bovis* in 351 broncho-alveolar lavage fluids (BALF) from calf farms in Algeria [42]. Thus, the extensive use of the *real-time* PCR could unlock new opportunities for the control of diseases caused by *M. bovis* provided the costs involved are cut. Developing new qPCR assays remains one of the attempts towards cost reduction. Chauhan and colleagues (2021) developed a multiplex qPCR to simultaneously detect *M. bovis*, *A. laidlawii*, and several Mycoplasma species, like *M. californicum*, *M. bovigenitalium*, *M. canadense*, *M. arginini*, and *M. alkalescens*. This assay, developed to target the *16S rRNA* gene of *Mycoplasma*, *rpoB* gene of *M. bovis*, and the *16S-23S rRNA* intergenic transcribed spacer (ITS) region of *A. laidlawii*, could detect and distinguish *M. bovis* from other prevalent Mycoplasma spp. and the non-pathogenic *A. laidlawii* in the milk samples collected from California dairy farms. However, sample preparation involving a *QIAGEN* DNeasy Blood and Tissue Lysis kit could potentially soar the cost of running such assays [139]. 

#### 3.3.3. Recombinase Polymerase Amplification

Interferences from proteins, fats, and ions greatly hinder the quantitative detection of bacteria using real-time PCRs [140,141]. One of the remedies is protease pre-treatment prior to direct detection. Rossetti and colleagues [142] developed a real-time PCR assay targeting the *uvrC* gene to directly detect *M. bovis* from milk and tissue samples with highly reduced interference. Such an approach sheds light on the efforts towards the development of robust and effective testing, which is currently lacking. Simple yet robust, the recombinase polymerase amplification (RPA) technique is yet another promising isothermal DNA amplifying assay with reduced external interference for possible rapid field-applicable tests. 

By targeting *uvrC* genes, RPA directly detected 36.9%. of *M. bovis* genes after 15 min incubation at 39 °C and 5 min visualization without any interference. The 65 milk samples for this validation were from the eight different dairy farms in Baoding and Hengshui, Hebei Province, China [52]. In the same way, RPA was combined with a lateral flow dipstick (LFD), and the assay successfully detected *M. bovis* DNA in 30 min at 39 °C with a detection limit of 20 copies per reaction when compared to a real-time quantitative PCR (qPCR) assay [71]. Such findings open new frontiers for the exploration of a simple and cost-effective alternative to the real-time PCR.

#### 3.3.4. Loop-Mediated Isothermal Amplification 

Just like RPA, another potential pen-side test for the detection of *M. bovis* is the loop-mediated isothermal amplification (LAMP) assay [60,63,66,76,88,143], though extensive validation for its reliability awaits. As a simple and cost-effective assay, LAMP is a rapid test with a reaction taking less than 2 h. Moreover, there is no need to have expensive laboratory equipment, as a single temperature is required [144]. Accordingly, LAMP is perceived as a potential cheap diagnostic tool. However, high background signals of some assays, vulnerability to cross-contamination/DNA carryover, and the complex primer design may compromise the specificity, sensitivity, and simplicity of the assay, respectively [145,146]. 

To reduce background signals and cross-contamination, various DNA purification kits have been employed. They include the *MoBio* DNA extraction kit [63], *QIAGEN DNeasy* Blood and Tissue kit [66], *TIANamp* Genomic DNA kit [143], and Procedure for UltraRapid Extraction (PURE) kit [60]. However, such interventions attract additional costs to the technique, making it lose its cost-saving attribute. 

#### 3.3.5. Pulse Field Gel Electrophoresis 

During pulse field gel electrophoresis (PFGE), *M. bovis* genomic DNA is first extracted and then digested using a restriction enzyme. Later, the digested products can be assessed on an agarose gel by subjecting it to an electric field that periodically changes direction to aid the separation of the larger DNA fragments [19]. Through this technique, *M. bovis* was fingerprinted in 34.0% (n = 151) of infected milk samples and mucosal swabs of lactating cows [89]. Similarly, PFGE detected *M.bovis* in French calf feedlots [86] and Danish cattle [101], among others. While PFGE can identify bacteria up to the strain level, the technique is time-consuming and requires specialized skills. 

### 3.4. Mass Spectrometry

Following the cultural isolation, a more rapid technique of matrix-assisted laser desorption/ionization time-of-flight mass spectrometry (MALDI-TOF MS) can be applied to detect *M. bovis* [147]. The principal strength of this technique is based on its ability to only detect viable bacteria, implying the animal has active rather than historic infections [148]. It is worth noting that, for rapid and effective detection of *M. bovis* using MALDI-TOF MS in routine veterinary laboratories, culture enrichment is encouraged. Enrichment enhanced the identification of 38.0% of *M. bovis* from 100 bronchoalveolar lavage fluid (BALF) after 72 h of enrichment [54]. Likewise, McDaniel and Derscheid (2021), accurately identified all eight isolates of *M. bovis* using *M. arginini* and *M. alkalescens* as controls to evaluate the specificity of MALDI-TOF MS. The isolates were first cultured in pleuropneumonia-like organism (PPLO) broth with horse serum (University of California-Davis, Davis, CA) 4 days prior to use [51]. In the most recent attempt, Thompson et al. (2023) combined MALDI-TOF with machine learning as an alternative diagnostic tool to detect the high somatic cell count (SCC) and subclinical mastitis in dairy herds around Texas, USA. Their study involving 100 milk samples showed a high sensitivity and specificity [38]. As much as the technique is reliable, rapid, and cost-effective for routine identification of unknown *Mycoplasma* isolates, it suffers from the limitations of the culture technique. Additionally, MALDI-TOF MS is a recent technique, and only a limited library for *Mycoplasma* species exists. 

## 4. Conclusions and Future Perspectives

The present study systematically reviewed several *M. bovis* mastitis diagnostic approaches. The bacterial culture method requires a specialized laboratory, technical staff, and time-consuming procedures. However, bacterial cultures provide discrete colony isolates for DNA-based and mass spectrometry diagnostics. On the other hand, serological techniques are faster than cultures but have low specificity due to cross-reactivity. The 2–3 weeks seroconversion period also increases the chances of false negative results. Hence, it is advisable to complement serology with other DNA-based techniques to achieve the accurate detection of early and chronic *M. bovis*. DNA-based assays, especially PCRs, are the most used technique due to their quick and accurate results, allowing for timely intervention. As much as the PCR has the potential to overcome the limitations of cultures and serology, it is costly and requires specialized equipment that limits its use in point-of-care field settings. While combining two techniques for diagnosing *M. bovis* is recommended, isothermal amplification technology (IAT) has shown higher sensitivity and specificity as a stand-alone technique with results obtained within two hours. Collectively, isothermal techniques just like any new diagnostic test should be validated before clinical use.

## Figures and Tables

**Table 1:** Recent techniques reported in the detection of *M. bovis*. Sixty-seven diagnostic studies utilizing various biological samples were explored. The included studies were those published between January 2000 and April 2023.

S/No.	Biological Sample	Technique	Detection Unit	*M. bovis* Positive Samples	Year	Country	References
1	Milk	MALDI-TOF MS and ML	Viable germs	<63%	2023	USA	[38]
2	Blood, Milk and Conjunctival fluids	ELISA and PCR	Antibodies and *M. bovis* genes	25.2%, 24.4% and 26.6%, 23.9%	2022	Netherlands	[39]
3	Synovial and lung tissue	IHC, PCR and Culture	*16S-23S rRNA ITS* gene	DAH	2022	Argentina	[40]
4	Nasopharyngeal swabs	PCR	*mb-mp81* genes	8.3%	2022	Egypt	[41]
5	Bronchoalveolar lavage fluid and serum	ELISA and Real-time PCR	Antibodies and *polC* genes	69.0% (241/351) and 58.0% (102/351)	2022	Algeria	[42]
6	Bulk tank milk	ELISA	Antibodies	44.7% (588/1313)	2022	Ireland	[43]
7	Serum	ELISA	*M. bovis* antibodies	7.2% (13/180)	2022	Sudan	[44]
8	Nasal swabs, tracheal tissues and swabs	Culture and PCR	Viable germ and *gyrA, parC* genes	61%	2022	Egypt	[45]
9	Nasal swabs	Multiplex PCR	*16S rRNA, oppD* and *oppF* genes	21.1%	2022	Mexico	[46]
10	Lung swabs	PCR	*ma-mp81* gene	86.9% (20/23)	2021	Spain	[47]
11	Nasal, trachea swabs and lung tissue	Culture and PCR	Viable germ and *16S rRNA* gene	67.5% (206/305) and 35.0% (7/20)	2021	Egypt	[48]
12	Bulk tank milk	Multiplex PCR	*M. bovis* genes	7.9% (3/35)	2021	Argentina	[49]
13	Serum	ELISA	Antibodies	48.7% (467/959)	2021	China	[50]
14	Isolates	MALDI-TOF MS and culture	Viable germs	CAD	2021	USA	[51]
15	Milk	Real-time PCR and LFS-RPA	*UvrC* genes	36.9% (24/65)	2021	China	[52]
16	Serum	ELISA	*M. bovis* antibodies	62.3% (249/400)	2021	Brazil	[53]
17	Nasal swabs	Real-time PCR and Cultures	*polC* genes	51% (59/251) and 52% (60/251)	2020	France	[8]
18	Bronchoalveolar lavage fluid	MALDI-TOF MS and Culture	Viable germ	38/100 (38%)	2020	Belgium	[54]
19	Milk	Real-time PCR	*uvrC* genes	1.1% (13/1166)	2020	Brazil	[55]
20	Vaginal fluid	PCR and ELISA	*16S-23S rRNA ITS* gene and antibody	0.2% (1/629)	2020	Australia	[56]
21	Blood and milk	ELISA, PCR and culture	*M. bovis* antibodies, genes and germ	DAH	2020	Australia	[57]
22	Pulmonary tissue	IHC	*M. bovis* antigens	91.4% (32/35)	2020	Brazil	[58]
23	Tracheobronchial lavage, nasal and milk	PCR and culture	*M. bovis* genes and genomes	DAH	2020	Estonia	[59]
24	Milk	PURE-LAMP	*M. bovis* genes	57.0%-97.0%	2020	Japan	[60]
25	Lung tissue	PCR	*M. bovis* genes	86.5%	2019	Iraq	[61]
26	Nasopharyngeal lavage, nasal and serum	Culture, ELISA and PCR	*M. bovis* germ, antibodies and *oppD*	DAH	2019	Finland	[62]
27	Culture suspensions	LAMP and Real-time PCR	*oppD* and *gltX* genes	100% (13/13) and 87.5% (14/16)	2019	USA	[63]
28	Lung tissues	IHC, Culture and PCR	*M. bovis* antigens and genes	18.8%	2019	India	[64]
29	Mammary tissue	DFAT	*M. bovis* antigens	23.3% (28/120)	2019	Turkey	[65]
30	Milk	LAMP	*UvrC*, *16S rRNA* and *gryB* genes	100% (30/30), 96.6% (29/30) and 86.6% (26/30)	2018	USA	[66]
31	Lung tissue and milk	Real-time PCR	*uvrC* gene	2% (1/51)	2018	India	[67]
32	Milk	PCR and culture	*oppD* gene and viable germ	10.0% (3/30) and 8.9% (4/45)	2018	Finland	[16]
33	Synovial fluid and lung tissue	PCR	*vsP* genes	27.3%	2018	Jordan	[68]
34	Serum and milk	ELISA	Antibodies	DAH	2018	Denmark	[69]
35	Semen and serum	PCR and ELISA	*16S-23S rRNA ITS* gene and antibody	3.4% and 46.0%	2018	Australia	[70]
36	Lung and nasal swab, join fluids and milk	RFLP-LFD	*uvrC, oppD*, and *oppF* genes	99.0%	2018	China	[71]
37	Serum	ELISA	Antibodies	9.9% (13/131)	2018	Serbia	[72]
38	Milk, serum, eye, and vaginal swabs	Culture and PCR	Viable germ and *16S rRNA* gene	23.0% (111/474) and 27.0% (102/474)	2017	Australia	[73]
39	Blood, eye, nose, and vaginal swabs	ELISA and PCR	Antibody and *16S-23S rRNA ITS* gene	93.8% (15/16) and 18.8% (3/16)	2017	Australia	[74]
40	Milk, BALF, lung, and synovial fluid	PCR	*M. bovis* genes	32.1%	2016	Turkey	[75]
41	Serum and nasopharyngeal swabs	Culture, ELISA, PCR, DGGE	*M. bovis* germ, antibodies, and genes	6.9% (49/713), 7.3% (52/713), 5.5% (39/713), 9.3% (66/713)	2016	Poland	[24]
42	Nasal swabs	LAMP	*OppD/F*	PWP	2016	Japan	[76]
43	Milk and nasal swab	Real-time PCR	*UvrC* genes	2.4% (18/742) and 31.9% (44/138)	2015	Switzerland	[77]
44	Blood	ELISA	Antibodies	19.5% (78/400)	2015	Nigeria	[78]
45	Milk	PCR	*M. bovis* genes	71.4% (10/14)	2015	Austria	[79]
46	Tracheal swabs and blood sera	PCR and ELISA	*mb-mp81* gene and antibodies	2.6% (16/127) and 35.4% (45/127)	2014	Turkey	[80]
47	Lung tissue	Culture and qPCR	Viable germ and *M. bovis* gene	19.3% (29/150) and 35.3% (53/150)	2014	Ireland	[81]
48	Milk	Culture, DFAT, and PCR	*M. bovis* germ, antibodies, and genes	DAH	2014	USA	[82]
49	Isolates	SDS-PAGE IB	*M.bovis* antigenic proteins	PWP	2014	India	[83]
50	Nasal	SDS-PAGE IB	*M.bovis* antigenic proteins	PWP	2013	India	[84]
51	Milk, semen, nasal, and vaginal discharge	PCR	*mbvF* genes	26.3% (101/384)	2012	India	[85]
52	Feedlot	PFGE	DNA fragments	MAH	2012	France	[86]
53	Milk	Culture and real-time PCR	Viable germ and *UvrC* genes	<0.1%	2011	France	[87]
54	Lung tissue, nasal, and trachea swabs	LAMP	*uvrC* genes	100% (6/6), 90% (46/51), 100% (2/2)	2011	China	[88]
55	Mucosal swabs and milk	PFGE, PCR, and culturing	DNA fragments	34.0% (54/151)	2010	USA	[89]
56	Bulk tank milk	Culture	Viable germs	7.0% (7/222)	2009	USA	[90]
57	Serum	ELISA	Antibodies	61.8% (139/225)	2008	Mexico	[91]
58	Joint fluids and lung tissues	PCR	*M. bovis* genes	DAH	2007	USA	[92]
59	Caseonecrotic lung tissue	Culture	Viable germs	98% (53/54)	2006	Canada	[93]
60	Lung tissue and milk	Real-time PCR	*16S rRNA* genes	(100%) 13/13 and 96.6% (28/29)	2005	Canada	[94]
61	Milk and nasal mucus	Multiplex PCR and culture	*mb-mp81* gene	MAH	2005	Italy	[95]
62	Milk, nasal, conjunctiva, and vaginal	Semi-nested PCR	*M. bovis* genes	27.5%, 30.0%, 12.5% and 2.5%	2003	Australia	[96]
63	Milk	IBT and Culture	*M. bovis* antigens	42.3% (55/130)	2002	Mexico	[97]
64	Serum	ELISA	*M. bovis* antibodies	7%	2002	France	[98]
65	Mucosal swabs	Nested PCR and Culture	*M. bovis* genes	49.1% (26/53)	2001	Germany	[99]
66	Bulk tank milk	Culture	Viable germs	7.0% (5/71)	2000	Chile	[100]
67	Lungs	PFGE	*M. bovis* DNA fragments	24.0%	2000	Denmark	[101]

LFS, Lateral Flow Strip; DAH, Detected Across Herds; RPA, Recombinase Polymerase Amplification; DGGE, Denaturing Gradient Gel Electrophoresis; PURE, Procedure for Ultra Extraction; PCR, Polymerase Chain Reaction; IHC, Immunohistochemistry; DFAT, Direct Fluorescent Antibody Technique; ELISA, Enzyme-linked Immunosorbent Assay; BALF, Bronchoalveolar Lavage Fluid; LAMP, Loop-mediated Isothermal Amplification; IG and F, Italy, Germany, and France; DFAT, Direct Fluorescent Antibody Test; CAD, Complete Accurate Detection; SDS-PAGE IB, Sodium Dodecyl-sulfate Polyacrylamide Gel Electrophoresis and Immunoblotting; PWP, Positive Without Proportion; PFGE, Pulse Field Gel Electrophoresis; IBT, Immunobinding Test; ML, Machine Learning.

## Data Availability

The original contributions presented in the study are included in the article.

## References

[1] Bradley A.J. (2002). Bovine Mastitis: An Evolving Disease. Vet. J..

[2] Chockalingam A., Zarlenga D.S., Bannerman D.D. (2007). Antimicrobial Activity of Bovine Bactericidal Permeability–Increasing Protein–Derived Peptides against Gram-Negative Bacteria Isolated from the milk of cows with clinical mastitis. Am. J. Veter. Res..

[3] Kirk J.H., Glenn K., Ruiz L., Smith E. (1997). Epidemiologic Analysis of Mycoplasma Spp Isolated from Bulk-Tank Milk Samples Obtained from Dairy Herds That Were Members of a Milk Cooperative. J. Am. Vet. Med. Assoc..

[4] Gioia G., Addis M.F., Santisteban C., Gross B., Nydam D.V., Sipka A.S., Virkler P.D., Watters R.D., Wieland M., Zurakowski M.J. (2021). Mycoplasma Species Isolated from Bovine Milk Collected from US Dairy Herds between 2016 and 2019. J. Dairy Sci..

[5] Ter Laak E., Noordergraaf J.H., Dieltjes R.P.J.W. (1992). Prevalence of Mycoplasmas in the Respiratory Tracts of Pneumonic Calves. J. Vet. Med. Ser. B.

[6] Walz P.H., Mullaney T.P., Render J.A., Walker R.D., Mosser T., Baker J.C. (1997). Otitis Media in Preweaned Holstein Dairy Calves in Michigan Due to Mycoplasma Bovis. J. Vet. Diagn. Investig..

[7] Langford E.V. (1975). Mycoplasma Species Recovered from the Reproductive Tracts of Western Canadian Cows. Canad. J. Comp. Med..

[8] Becker C.A.M., Ambroset C., Huleux A., Vialatte A., Colin A., Tricot A., Arcangioli M.A., Tardy F. (2020). Monitoring *Mycoplasma bovis* Diversity and Antimicrobial Susceptibility in Calf Feedlots Undergoing a Respiratory Disease Outbreak. Pathogens.

[9] Kirby F., Nicholas R. (1996). Isolation of *Mycoplasma bovis* from Bullocks’ Eyes. Vet. Rec..

[10] Krysak D.E. (2006). Chronic Pneumonia and Polyarthritis Syndrome in a Feedlot Calf. Can. Vet. J..

[11] Li Y., Zheng H., Liu Y., Jiang Y., Xin J., Chen W., Song Z. (2011). The Complete Genome Sequence of *Mycoplasma bovis* Strain Hubei-1. PLoS ONE.

[12] Wise K.S., Calcutt M.J., Foecking M.F., Röske K., Madupu R., Methé B.A. (2011). Complete Genome Sequence of *Mycoplasma bovis* Type Strain PG45 (ATCC 25523). Infect. Immun..

[13] Marcone C. (2014). Molecular Biology and Pathogenicity of Phytoplasmas. Ann. Appl. Biol..

[14] Fox L.K. (2012). Mycoplasma Mastitis. Causes, Transmission, and Control. Vet. Clin. North Am. Food Anim. Pract..

[15] Aebi M., Bodmer M., Frey J., Pilo P. (2012). Herd-Specific Strains of *Mycoplasma bovis* in Outbreaks of Mycoplasmal Mastitis and Pneumonia. Vet. Microbiol..

[16] Haapala V., Pohjanvirta T., Vähänikkilä N., Halkilahti J., Simonen H., Pelkonen S., Soveri T., Simojoki H., Autio T. (2018). Semen as a Source of *Mycoplasma bovis* Mastitis in Dairy Herds. Vet. Microbiol..

[17] Nicholas R.A.J., Fox L.K., Lysnyansky I. (2016). Mycoplasma Mastitis in Cattle: To Cull or Not to Cull. Vet. J..

[18] Nicholas R.A.J., Ayling R.D. (2003). Mycoplasma Bovis: Disease, Diagnosis, and Control. Res. Vet. Sci..

[19] McAuliffe L., Kokotovic B., Ayling R.D., Nicholas R.A.J. (2004). Molecular Epiderniological Analysis of *Mycoplasma bovis* Isolates from the United Kingdom Shows Two Genetically Distinct Clusters. J. Clin. Microbiol..

[20] Rosengarten R., Citti C. (1999). The Role of Ruminant Mycoplasmas in Systemic Infection. Mycoplasmas Rumin. Pathog. Diagn. Epidemiol. Mol. Genet..

[21] USDA (2018). Dairy 2014. Health and Management Practices on U.S. Dairy Operations 2014.

[22] Kusiluka L.J.M., Kokotovic B., Ojeniyi B., Friis N.F., Ahrens P. (2006). Genetic Variations among *Mycoplasma bovis* Strains Isolated from Danish Cattle. FEMS Microbiol. Lett..

[23] Dudek K., Szacawa E. (2020). *Mycoplasma bovis* Infections: Occurrence, Pathogenesis, Diagnosis and Control, Including Prevention and Therapy. Pathogens.

[24] Szacawa E., Szymańska-Czerwińska M., Niemczuk K., Dudek K., Bednarek D., Ayling R.D. (2016). Comparison of Serological, Molecular and Cultural Diagnostic Methods for the Detection of *Mycoplasma bovis* Infections in Cattle. Anim. Sci. Pap. Rep..

[25] Kumar A., Verma A.K., Rahal A. (2011). Mycoplasma Bovis, a Multi Disease Producing Pathogen: An Overview. Asian J. Anim. Vet. Adv..

[26] Motaung T.E., Petrovski K.R., Petzer I.M., Thekisoe O., Tsilo T.J. (2017). Importance of Bovine Mastitis in Africa. Anim. Heal. Res. Rev..

[27] Maunsell F.P., Woolums A.R., Francoz D., Rosenbusch R.F., Step D.L., Wilson D.J., Janzen E.D. (2011). *Mycoplasma bovis* Infections in Cattle. J. Vet. Intern. Med..

[28] Calcutt M.J., Lysnyansky I., Sachse K., Fox L.K., Nicholas R.A.J., Ayling R.D. (2018). Gap Analysis of *Mycoplasma bovis* Disease, Diagnosis and Control: An Aid to Identify Future Development Requirements. Transbound. Emerg. Dis..

[29] Parker A.M., Sheehy P.A., Hazelton M.S., Bosward K.L., House J.K. (2018). A Review of Mycoplasma Diagnostics in Cattle. J. Vet. Intern. Med..

[30] Gelgie A.E., Korsa M.G., Kerro Dego O. (2022). *Mycoplasma bovis* Mastitis. Curr. Res. Microb. Sci..

[31] Nicholas R.A.J. (2004). Recent Developments in the Diagnosis and Control of Mycoplasma Infections in Cattle. Medecin Veterinaire du Quebec.

[32] Moher D., Liberati A., Tetzlaff J., Altman D.G. (2009). Preferred Reporting Items for Systematic Reviews and Meta-Analyses: The PRISMA Statement. BMJ.

[33] Russell C.K., Gregory D.M. (2003). EBN Users’ Guide Evaluation of Qualitative Research Studies Clinical Scenario. Evid. Based Nurs..

[34] Munn Z., MClinSc S.M., Lisy K., Riitano D., Tufanaru C. (2015). Methodological Guidance for Systematic Reviews of Observational Epidemiological Studies Reporting Prevalence and Cumulative Incidence Data. Int. J. Evid. Based Healthc..

[35] Peters M.D.J., Godfrey C.M., Khalil H., McInerney P., Parker D., Soares C.B. (2015). Guidance for Conducting Systematic Scoping Reviews. Int. J. Evid. Based Healthc..

[36] Aromataris E., Fernandez R., Godfrey C.M., Holly C., Khalil H., Tungpunkom P. (2015). Summarizing Systematic Reviews: Methodological Development, Conduct and Reporting of an Umbrella Review Approach. Int. J. Evid. Based Healthc..

[37] Walsh D., Downe S. (2006). Appraising the Quality of Qualitative Research. Midwifery.

[38] Thompson J., Everhart Nunn S.L., Sarkar S., Clayton B. (2023). Diagnostic Screening of Bovine Mastitis Using MALDI-TOF MS Direct-Spotting of Milk and Machine Learning. Vet. Sci..

[39] Penterman P.M., Holzhauer M., van Engelen E., Smits D., Velthuis A.G.J. (2022). Dynamics of *Mycoplasma bovis* in Dutch Dairy Herds during Acute Clinical Outbreaks. Vet. J..

[40] Cantón G., Llada I., Margineda C., Urtizbiría F., Fanti S., Scioli V., Fiorentino M.A., Louge Uriarte E., Morrell E., Sticotti E. (2022). Mycoplasma Bovis-Pneumonia and Polyarthritis in Feedlot Calves in Argentina: First Local Isolation. Rev. Argent. Microbiol..

[41] Hashem Y.M., Mousa W.S., Abdeen E.E., Abdelkhalek H.M. (2022). Prevalence and Molecular Characterization of Mycoplasma Species, Pasteurella Multocida, and Staphylococcus Aureus Isolated from Calves with Respiratory Manifestations. Animals.

[42] Oucheriah Y., Heleili N., Colin A., Mottet C., Tardy F., Becker C.A.M. (2022). Prevalence of *Mycoplasma bovis* in Algeria and Characterisation of the Isolated Clones. Front. Vet. Sci..

[43] McAloon C.I., McAloon C.G., Tratalos J., O’Grady L., McGrath G., Guelbenzu M., Graham D.A., O’Keeffe K., Barrett D.J., More S.J. (2022). Seroprevalence of *Mycoplasma bovis* in Bulk Milk Samples in Irish Dairy Herds and Risk Factors Associated with Herd Seropositive Status. J. Dairy Sci..

[44] Onsa R.A.H., Aldeen H., Abdelrazig M., Kit E. (2022). *Mycoplasma bovis* Seroprevalence in Khartoum. Asian J. Res. Anim. Vet. Sci..

[45] Ammar A.M., Abd El-Hamid M.I., Mohamed Y.H., Mohamed H.M., Al-khalifah D.H.M., Hozzein W.N., Selim S., El-Neshwy W.M., El-Malt R.M.S. (2022). Prevalence and Antimicrobial Susceptibility of Bovine Mycoplasma Species in Egypt. Biology.

[46] Maya-Rodríguez L.M., Carrillo-Casas E.M., Rojas-Trejo V., Trigo-Tavera F., Miranda-Morales R.E. (2022). Prevalence of Three Mycoplasma Sp. by Multiplex PCR in Cattle with and without Respiratory Disease in Central Mexico. Trop. Anim. Health Prod..

[47] García-Galán A., Seva J., Gómez-Martín Á., Ortega J., Rodríguez F., García-Muñoz Á., De la Fe C. (2021). Importance and Antimicrobial Resistance of *Mycoplasma bovis* in Clinical Respiratory Disease in Feedlot Calves. Animals.

[48] Abd El-Tawab A., Elhofy F., Hassan N., Ramadan M. (2021). Identification and Genetic Characterization of Mycoplasma Species Affecting Respiratory System in Egyptian Cattle. Benha Vet. Med. J..

[49] Neder V.E., Amadio A.F., Calvinho L.F. (2021). Detection by Multiplex PCR of Mycoplasma Species Associated with Dairy Cattle in Argentina. Rev. Argent. Microbiol..

[50] Niu J., Li K., Pan H., Gao X., Li J., Wang D., Yan M., Xu Y., Sizhu S. (2021). Epidemiological Survey of *Mycoplasma bovis* in Yaks on the Qinghai Tibetan Plateau, China. Biomed Res. Int..

[51] McDaniel A.J., Derscheid R.J. (2021). MALDI-TOF Mass Spectrometry and High-Resolution Melting PCR for the Identification of *Mycoplasma bovis* Isolates. BMC Vet. Res..

[52] Li R., Wang J., Sun X., Liu L., Wang J., Yuan W. (2021). Direct and Rapid Detection of *Mycoplasma bovis* in Bovine Milk Samples by Recombinase Polymerase Amplification Assays. Front. Cell. Infect. Microbiol..

[53] Pires D.R., Morais A.C.N., Cunha N.C., Machado L.S., Barbosa L.F.C., Mendonça J.F.M., Balaro M.F.A., Santos J.P.C., Souza G.N., Barreto M.L. (2021). Proposal of an IELISA for *Mycoplasma bovis* Diagnosis in Dairy Cattle and Associated Risk Factors. Arq. Bras. Med. Vet. e Zootec..

[54] Bokma J., Van Driessche L., Deprez P., Haesebrouck F., Vahl M., Weesendorp E., Deurenberg R.H., Pardon B., Boyen F. (2020). Rapid Identification of *Mycoplasma bovis* Strains from Bovine Bronchoalveolar Lavage Fluid with Matrix-Assisted Laser. J. Clin. Microbiol..

[55] Salina A., Timenetsky J., Barbosa M.S., Azevedo C.M., Langoni H. (2020). Microbiological and Molecular Detection of *Mycoplasma bovis* in Milk Samples from Bovine Clinical Mastitis. Pesqui. Vet. Bras..

[56] Hazelton M.S., Morton J.M., Bosward K.L., Sheehy P.A., Parker A.M., Dwyer C.J., Niven P.G., House J.K. (2020). Mycoplasma Species in Vaginas of Dairy Cows before and after Exposure to Bulls and Their Association with Conception. J. Dairy Sci..

[57] Hazelton M.S., Morton J.M., Parker A.M., Sheehy P.A., Bosward K.L., Malmo J., House J.K. (2020). Whole Dairy Herd Sampling to Detect Subclinical Intramammary *Mycoplasma bovis* Infection after Clinical Mastitis Outbreaks. Vet. Microbiol..

[58] Oliveira T.E.S., Pelaquim I.F., Flores E.F., Massi R.P., Valdiviezo M.J.J., Pretto-Giordano L.G., Alfieri A.A., Saut J.P.E., Headley S.A. (2020). *Mycoplasma bovis* and Viral Agents Associated with the Development of Bovine Respiratory Disease in Adult Dairy Cows. Transbound. Emerg. Dis..

[59] Timonen A.A.E., Autio T., Pohjanvirta T., Häkkinen L., Katholm J., Petersen A., Mõtus K., Kalmus P. (2020). Dynamics of the Within-Herd Prevalence of *Mycoplasma bovis* Intramammary Infection in Endemically Infected Dairy Herds. Vet. Microbiol..

[60] Itoh M., Hirano Y., Yamakawa K., Yasutomi I., Kuramoto K., Furuoka M., Yamada K. (2020). Combination of Procedure for Ultra Rapid Extraction (Pure) and Loop-Mediated Isothermal Amplification (Lamp) for Rapid Detection of *Mycoplasma bovis* in Milk. J. Vet. Med. Sci..

[61] Hamad M.A., AL-Jumaa Z.M., Al-Aalim A.M., Jaber Mayahi M.T. (2019). Detection of *Mycoplasma bovis* in Pneumonic Calves. J. Pure Appl. Microbiol..

[62] Vähänikkilä N., Pohjanvirta T., Haapala V., Simojoki H., Soveri T., Browning G.F., Pelkonen S., Wawegama N.K., Autio T. (2019). Characterisation of the Course of *Mycoplasma bovis* Infection in Naturally Infected Dairy Herds. Vet. Microbiol..

[63] Appelt S., Aly S.S., Tonooka K., Glenn K., Xue Z., Lehenbauer T.W., Marco M.L. (2019). Development and Comparison of Loop-Mediated Isothermal Amplification and Quantitative Polymerase Chain Reaction Assays for the Detection of *Mycoplasma bovis* in Milk. J. Dairy Sci..

[64] Goswami P., Banga H.S., Mahajan V. (2019). Pathological Description of Naturally Occurring *Mycoplasma bovis* Associated Pneumonia in Bovine Calves. Indian J. Anim. Res..

[65] Altun S., Ozdemir S. (2019). Detection of *Mycoplasma bovis* Infection in Cattle Mammary Tissue by Immunofluorescence and QRT-PCR Methods. Kocatepe Vet. J..

[66] Ashraf A., Imran M., Yaqub T., Tayyab M., Shehzad W., Mingala C.N., Chang Y.F. (2018). Development and Validation of a Loop-Mediated Isothermal Amplification Assay for the Detection of *Mycoplasma bovis* in Mastitic Milk. Folia Microbiol..

[67] Behera S., Rana R., Gupta P.K., Kumar D., Sonal, Rekha V., Arun T.R., Jena D. (2018). Development of Real-Time PCR Assay for the Detection of *Mycoplasma bovis*. Trop. Anim. Health Prod..

[68] Hananeh W.M., Al Momani W.M., Ababneh M.M., Abutarbush S.M. (2018). *Mycoplasma bovis* Arthritis and Pneumonia in Calves in Jordan: An Emerging Disease. Vet. World.

[69] Petersen M.B., Pedersen J., Holm D.L., Denwood M., Nielsen L.R. (2018). A Longitudinal Observational Study of the Dynamics of *Mycoplasma bovis* Antibodies in Naturally Exposed and Diseased Dairy Cows. J. Dairy Sci..

[70] Hazelton M.S., Morton J.M., Bosward K.L., Sheehy P.A., Parker A.M., Dwyer C.J., Niven P.G., House J.K. (2018). Isolation of Mycoplasma Spp. and Serological Responses in Bulls Prior to and Following Their Introduction into *Mycoplasma bovis*-Infected Dairy Herds. J. Dairy Sci..

[71] Zhao G., Wang H., Hou P., Xia X., He H. (2018). A Lateral Flow Dipstick Combined with Reverse Transcription Recombinase Polymerase Amplification for Rapid and Visual Detection of the Bovine Respirovirus 3. Mol. Cell. Probes.

[72] Vojinović D., Zdravković N., Prodanović R., Vujanac I., Nedić S., Giadinis N.D., Panousis N., Manić M., Bugarski D., Palamarević M. (2018). Seroprevalence of *Mycoplasma bovis* in Grazing Dairy Cows from Five Different Areas in Serbia. J. Hell. Vet. Med. Soc..

[73] Parker A.M., House J.K., Hazelton M.S., Bosward K.L., Sheehy P.A. (2017). Comparison of Culture and a Multiplex Probe PCR for Identifying Mycoplasma Species in Bovine Milk, Semen and Swab Samples. PLoS ONE.

[74] Hazelton M.S., Sheehy P.A., Bosward K.L., Parker A.M., Morton J.M., Dwyer C.J., Niven P.G., House J.K. (2017). Short Communication: Shedding of *Mycoplasma bovis* and Antibody Responses in Cows Recently Diagnosed with Clinical Infection. J. Dairy Sci..

[75] Sayin Z., Sakmanoğlu A., Uçan U.S., Uslu A., Hadimli H.H., Aras Z., Özdemir Ö., Erganis O. (2016). Mycoplasma Infections in Dairy Cattle Farms in Turkey. Turkish J. Vet. Anim. Sci..

[76] Higa Y., Uemura R., Yamazaki W., Goto S., Goto Y., Sueyoshi M. (2016). An Improved Loop-Mediated Isothermal Amplification Assay for the Detection of Mycoplasma Bovis. J. Vet. Med. Sci..

[77] Aebi M., van den Borne B.H.P., Raemy A., Steiner A., Pilo P., Bodmer M. (2015). *Mycoplasma bovis* Infections in Swiss Dairy Cattle: A Clinical Investigation. Acta Vet. Scand..

[78] Francis M.I., Raji M.A., Kazeem H.M., Suleiman M.M. (2015). ELISA-Based Serological Survey of *Mycoplasma bovis* in Cattle in Three Local Government Areas in Adamawa State, Nigeria. J. Adv. Vet. Anim. Res..

[79] Pothmann H., Spergser J., Elmer J., Prunner I., Iwersen M., Klein-Jöbstl D., Drillich M. (2015). Severe *Mycoplasma bovis* Outbreak in an Austrian Dairy Herd. J. Vet. Diagn. Investig..

[80] Akan M., Babacan O., Torun E., Müştak H.K., Öncel T. (2014). Diagnosis of *Mycoplasma bovis* Infection in Cattle by ELISA and PCR. Kafkas Univ. Vet. Fak. Derg..

[81] Bell C.J., Blackburn P., Elliott M., Patterson T.I.A.P., Ellison S., Lahuerta-Marin A., Ball H.J. (2014). Investigation of Polymerase Chain Reaction Assays to Improve Detection of Bacterial Involvement in Bovine Respiratory Disease. J. Vet. Diagn. Investig..

[82] Murai K., Lehenbauer T.W., Champagne J.D., Glenn K., Aly S.S. (2014). Cost-Effectiveness of Diagnostic Strategies Using Quantitative Real-Time PCR and Bacterial Culture to Identify Contagious Mastitis Cases in Large Dairy Herds. Prev. Vet. Med..

[83] Kumar A., Srivastava N.C., Singh V.P., Sunder J. (2014). Electrophoretic Analysis of Indian Isolates of Mycoplasma Agalactiae and *Mycoplasma bovis* by SDS-PAGE and Immunoblotting. Vet. Med. Int..

[84] Kumar A., Srivastava N.C., Uphadhayay D.D., Sansthan A. (2013). Identification of Potential Diagnostic and Protective Antigen of Mycoplasma Bovis. Indian J. Camp. Microbial. Immunol. Infect. Dis..

[85] Jain U., Verma A.K., Pal B.C. (2012). PCR Based Detection of *Mycoplasma bovis* from Bovine Clinical Specimens. Indian Vet. J..

[86] Arcangioli M.A., Aslan H., Tardy F., Poumarat F., Le Grand D. (2012). The Use of Pulsed-Field Gel Electrophoresis to Investigate the Epidemiology of *Mycoplasma bovis* in French Calf Feedlots. Vet. J..

[87] Arcangioli M.A., Chazel M., Sellal E., Botrel M.A., Bézille P., Poumarat F., Calavas D., Le Grand D. (2011). Prevalence of *Mycoplasma bovis* Udder Infection in Dairy Cattle: Preliminary Field Investigation in Southeast France. N. Z. Vet. J..

[88] Bai Z., Shi L., Hu C., Chen X., Qui J., Ba X., Peng Q., Chen Y., Chen H., Guo A. (2011). Development of a Loop-Mediated Isothermal Amplification Assay for Sensitive and Rapid Detection of *Mycoplasma bovis*. Afr. J. Biotechnol..

[89] Punyapornwithaya V., Fox L.K., Hancock D.D., Gay J.M., Alldredge J.R. (2010). Association between an Outbreak Strain Causing *Mycoplasma bovis* Mastitis and Its Asymptomatic Carriage in the Herd: A Case Study from Idaho, USA. Prev. Vet. Med..

[90] Wilson D.J., Goodell G., Justice-Allen A., Smith S.T. (2009). Herd-Level Prevalence of Mycoplasma Spp Mastitis and Characteristics of Infected Dairy Herds in Utah as Determined by a Statewide Survey. J. Am. Vet. Med. Assoc..

[91] Núñez D.C., Morales S.E., Martínez M.J.J., Hernández A.L. (2008). Detection of Subclinical Bovine Mastitis Caused by Mycoplasmosis by Indirect ELISA Test and Isolation. Vet. Mex..

[92] Wilson D.J., Skirpstunas R.T., Trujillo J.D., Cavender K.B., Bagley C.V., Harding R.L. (2007). Unusual History and Initial Clinical Signs of *Mycoplasma bovis* Mastitis and Arthritis in First-Lactation Cows in a Closed Commercial Dairy Herd. J. Am. Vet. Med. Assoc..

[93] Gagea M.I., Bateman K.G., Shanahan R.A., Van Dreumel T., McEwen B.J., Carman S., Archambault M., Caswell J.L. (2006). Naturally Occurring Mycoplasma Bovis-Associated Pneumonia and Polyarthritis in Feedlot Beef Calves. J. Vet. Diagn. Investig..

[94] Cai H.Y., Bell-Rogers P., Parker L., Prescott J.F. (2005). Development of a Real-Time PCR for Detection of *Mycoplasma bovis* in Bovine Milk and Lung Samples. J. Vet. Diagn. Investig..

[95] Foddai A., Idini G., Fusco M., Rosa N., De La Fe C., Zinellu S., Corona L., Tola S. (2005). Rapid Differential Diagnosis of Mycoplasma Agalactiae and *Mycoplasma bovis* Based on a Multiplex-PCR and a PCR-RFLP. Mol. Cell. Probes.

[96] Hayman B., Hirst R. (2003). Development of a Semi-Nested PCR for the Improved Detection of *Mycoplasma bovis* from Bovine Milk and Mucosal Samples. Vet. Microbiol..

[97] Infante F., Infante F., Flores-Guitiérrez G.H. (2002). Improved Immunobinding Test Using Monoclonal Antibodies for Detection of *Mycoplasma bovis* in Milk. Can. J. Vet. Res..

[98] Le Grand D., Bézille P., Calavas D., Poumarat F., Brank M., Citti C., Rosengarten R. (2002). Serological Prevalence of *Mycoplasma bovis* Infection in Suckling Beef Cattle in France. Vet. Rec..

[99] Pinnow C.C., Butler J.A., Sachse K., Hotzel H., Timms L.L., Rosenbusch R.F. (2001). Detection of *Mycoplasma bovis* in Preservative-Treated Field Milk Samples. J. Dairy Sci..

[100] Sickles S.A., Kruze J., González R.N. (2000). Detection of *Mycoplasma bovis* in Bulk Tank Milk Samples from Herds in Southern Chile. Arch. Med. Vet..

[101] Kusiluka L.J.M., Ojeniyi B., Friis N.F. (2000). Increasing Prevalence of *Mycoplasma bovis* in Danish Cattle. Acta Vet. Scand..

[102] Sachse K., Pfützner H., Hotzel H., Demuth B., Heller M., Berthold E. (1993). Comparison of Various Diagnostic Methods for the Detection of *Mycoplasma bovis*. Rev. Sci. Tech..

[103] Punyapornwithaya V., Fox L.K., Gay G.M., Hancock D.D., Alldredge J.R. (2009). The Effect of Centrifugation and Resuspension on the Recovery of Mycoplasma Species from Milk. J. Dairy Sci..

[104] NMC (1990). Microbial Procedures for the Diagnosis of Bovine Udder Infection.

[105] Ismael A.B., Hassan M., Mostafa S.A., Nassan M.A., Mohamed E.H. (2016). Development of a Second-Generation Vaccine against Mycoplasmosis: Preparation of a Fraction Candidate from *Mycoplasma bovis* and Its Evaluation as a Vaccine Department of Animal Medicine, Faculty of Veterinary Medicine, Department of Medical Microbiolog. Glob. Vet..

[106] Furr M.P., Miles R., Nicholas A.R. (1998). Cultivation of Ureaplasmas. Methods in Molecular Biology.

[107] Pfützner H., Sachse K. (1996). *Mycoplasma bovis* as an Agent of Mastitis, Pneumonia, Arthritis and Genital Disorders in Cattle. OIE Rev. Sci. Tech..

[108] Poumarat F., Longchambon D., Martel J.L. (1992). Application of Dot Immunobinding on Membrane Filtration (MF Dot) to the Study of Relationships within “M. Mycoides Cluster” and within “Glucose and Arginine-Negative Cluster” of Ruminant Mycoplasmas. Vet. Microbiol..

[109] Hoelzle K., Ade J., Hoelzle L.E. (2020). Persistence in Livestock Mycoplasmas—A Key Role in Infection and Pathogenesis. Curr. Clin. Microbiol. Rep..

[110] Boonyayatra S., Fox L.K., Gay J.M., Sawant A., Besser T.E. (2012). Discrimination between Mycoplasma and Acholeplasma Species of Bovine Origin Using Digitonin Disc Diffusion Assay, Nisin Disc Diffusion Assay, and Conventional Polymerase Chain Reaction. J. Vet. Diagn. Investig..

[111] Gioia G., Werner B., Nydam D.V., Moroni P. (2016). Validation of a Mycoplasma Molecular Diagnostic Test and Distribution of Mycoplasma Species in Bovine Milk among New York State Dairy Farms. J. Dairy Sci..

[112] Byrne W.J., Ball H.J., Brice N., McCormack R., Baker S.E., Ayling R.D., Nicholas R.A.J. (2000). Application of an Indirect ELISA to Milk Samples to Identify Cows with *Mycoplasma bovis* Mastitis. Vet. Rec..

[113] Adegboye D.S., Halbur P.G., Cavanaugh D.L., Werdin R.E., Chase C.C.L., Miskimins D.W., Rosenbusch R.F. (1995). Immunohistochemical and Pathological Study of Mycoplasma Bovis-Associated Lung Abscesses in Calves. J. Vet. Diagn. Investig..

[114] Mayes B., Rupprecht C.E. (2015). Direct Fluorescent Antibody Test for Rabies Diagnosis.

[115] Infante Martinez F., Jasper D.E., Stott J.L., Cullor J.S., Dellinger J.D. (1990). Immunobinding Assay for Detection of *Mycoplasma bovis* in Milk. Can. J. Vet. Res..

[116] Uhaa I., Riemann H., Thurmond M., Franti C. (1990). The Use of the Enzyme-Linked Immunosorbent Assay (ELISA) in Serological Diagnosis of *Mycoplasma bovis* in Dairy Cattle. Vet. Res. Commun..

[117] Wawegama N.K., Browning G.F., Kanci A., Marenda M.S., Markham P.F. (2014). Development of a Recombinant Protein-Based Enzyme-Linked Immunosorbent Assay for Diagnosis of *Mycoplasma bovis* Infection in Cattle. Clin. Vaccine Immunol..

[118] Miller J.J., Levinson S.S., Eleftherios D., Theodore C. (1996). Interferences in Immunoassays. Immunoassay.

[119] Sekiya M., Zintl A., Doherty M.L. (2013). Bulk Milk ELISA and the Diagnosis of Parasite Infections in Dairy Herds: A Review. Ir. Vet. J..

[120] Rodrigues A.M., Fernandes G.F., Araujo L.M., Della Terra P.P., dos Santos P.O., Pereira S.A., Schubach T.M.P., Burger E., Lopes-Bezerra L.M., de Camargo Z.P. (2015). Proteomics-Based Characterization of the Humoral Immune Response in Sporotrichosis: Toward Discovery of Potential Diagnostic and Vaccine Antigens. PLoS Negl. Trop. Dis..

[121] Alberti A., Addis M.F., Chessa B., Cubeddu T., Profiti M., Rosati S., Ruiu A., Pittau M. (2006). Molecular and Antigenic Characterization of a *Mycoplasma bovis* Strain Causing an Outbreak of Infectious Keratoconjunctivitis. J. Vet. Diagn. Investig..

[122] Flores-Gutiérrez G.H., Infante F., Salinas-Meléndez J.A., Thomas C.B., Estrada-Bellmann P.C., Briones-Encinia F. (2009). Development of a Rapid Immunobinding Test for *Mycoplasma bovis* Cultural Isolates in the Genital Tract of Heifers. Ital. J. Anim. Sci..

[123] Ligasová A., Vydržalová M., Buriánová R., Brůčková L., Večeřová R., Janoštáková A., Koberna K. (2019). A New Sensitive Method for the Detection of Mycoplasmas Using Fluorescence Microscopy. Cells.

[124] Hirose K., Kawasaki Y., Kotani K., Tanaka A., Abiko K., Ogawa H. (2001). Detection of Mycoplasma in Mastitic Milk by PCR Analysis and Culture Method. J. Vet. Med. Sci..

[125] Tang J., Hu M., Lee S., Roblin R. (2000). A Polymerase Chain Reaction Based Method for Detecting Mycoplasma/Acholeplasma Contaminants in Cell Culture. J. Microbiol. Methods.

[126] McAuliffe L., Ellis R.J., Lawes J.R., Ayling R.D., Nicholas R.A.J. (2005). 16S RDNA PCR and Denaturing Gradient Gel Electrophoresis; a Single Generic Test for Detecting and Differentiating Mycoplasma Species. J. Med. Microbiol..

[127] Schnee C., Schulsse S., Hotzel H., Ayling R.D., Nicholas R.A.J., Schubert E., Heller M., Ehricht R., Sachse K. (2012). A Novel Rapid Dna Microarray Assay Enables Identification of 37 Mycoplasma Species and Highlights Multiple Mycoplasma Infections. PLoS ONE.

[128] Biddle M.K., Fox L.K., Hancock D.D. (2003). Patterns of Mycoplasma Shedding in the Milk of Dairy Cows with Intramammary Mycoplasma Infection. J. Am. Vet. Med. Assoc..

[129] Subramaniam S., Bergonier D., Poumarat F., Capaul S., Schlatter Y., Nicolet J., Frey J. (1998). Species Identification of *Mycoplasma bovis* and Mycoplasma Agalactiae Based on the UvrC Genes by PCR. Mol. Cell. Probes.

[130] Xu W., Shang Y. (2016). The Detection Techniques of Genetically Modified Organisms. Genetically Modified Organisms in Food.

[131] Chifiriuc M.C., Gheorghe I., Czobor I., Florea D.A., Mateescu L., Caplan M.E., Caplan D.M., Lazar V., Grumezescu A.M. (2017). Advances in Molecular Biology Based Assays for the Rapid Detection of Food Microbial Contaminants. Food Preservation.

[132] Königsson M.H., Bölske G., Johansson K.E. (2002). Intraspecific Variation in the 16S RRNA Gene Sequences of Mycoplasma Agalactiae and *Mycoplasma bovis* Strains. Vet. Microbiol..

[133] Carr J., Williams D.G., Hayden R.T., Wayne W.G., Robert M.N., Charles M.S., Frederick L.K. (2010). Molecular Detection of Multiple Respiratory Viruses. Molecular Diagnostics.

[134] Green M.R., Sambrook J. (2019). Nested Polymerase Chain Reaction (PCR). Cold Spring Harb. Protoc..

[135] Kralik P., Ricchi M. (2017). A Basic Guide to Real Time PCR in Microbial Diagnostics: Definitions, Parameters, and Everything. Front. Microbiol..

[136] Koskinen M.T., Holopainen J., Pyörälä S., Bredbacka P., Pitkälä A., Barkema H.W., Bexiga R., Roberson J., Sølverød L., Piccinini R. (2009). Analytical Specificity and Sensitivity of a Real-Time Polymerase Chain Reaction Assay for Identification of Bovine Mastitis Pathogens. J. Dairy Sci..

[137] Koskinen M.T., Wellenberg G.J., Sampimon O.C., Holopainen J., Rothkamp A., Salmikivi L., van Haeringen W.A., Lam T.J.G.M., Pyörälä S. (2010). Field Comparison of Real-Time Polymerase Chain Reaction and Bacterial Culture for Identification of Bovine Mastitis Bacteria. J. Dairy Sci..

[138] Pansri P., Katholm J., Krogh K.M., Aagaard A.K., Schmidt L.M.B., Kudirkiene E., Larsen L.E., Olsen J.E. (2020). Evaluation of Novel Multiplex QPCR Assays for Diagnosis of Pathogens Associated with the Bovine Respiratory Disease Complex. Vet. J..

[139] Chauhan K., Aly S.S., Lehenbauer T.W., Tonooka K.H., Glenn K., Rossitto P., Marco M.L. (2021). Development of a Multiplex QPCR Assay for the Simultaneous Detection of Mycoplasma Bovis, Mycoplasma Species, and Acholeplasma Laidlawii in Milk. PeerJ.

[140] Acharya K.R., Dhand N.K., Whittington R.J., Plain K.M. (2017). PCR Inhibition of a Quantitative PCR for Detection of Mycobacterium Avium Subspecies Paratuberculosis DNA in Feces: Diagnostic Implications and Potential Solutions. Front. Microbiol..

[141] Kuffel A., Gray A., Daeid N.N. (2021). Impact of Metal Ions on PCR Inhibition and RT-PCR Efficiency. Int. J. Legal Med..

[142] Rossetti B.C., Frey J., Pilo P. (2010). Direct Detection of *Mycoplasma bovis* in Milk and Tissue Samples by Real-Time PCR. Mol. Cell. Probes.

[143] Song Q., Wang L., Fang R., Khan M.K., Zhou Y., Zhao J. (2012). Detection of Mycoplasma Wenyonii in Cattle and Transmission Vectors by the Loop-Mediated Isothermal Amplification (LAMP) Assay. Trop. Anim. Health Prod..

[144] Li Y., Fan P., Zhou S., Zhang L. (2017). Loop-Mediated Isothermal Amplification (LAMP): A Novel Rapid Detection Platform for Pathogens. Microb. Pathog..

[145] Wong Y., Othman Y., Radu S., Chee H. (2017). Loop-mediated isothermal amplification (LAMP): A versatile technique for detection of micro-organisms. J. Appl. Microbiol..

[146] Lau H.Y., Botella J.R. (2017). Advanced DNA-Based Point-of-Care Diagnostic Methods for Plant Diseases Detection. Front. Plant Sci..

[147] Randall L.P., Lemma F., Koylass M., Rogers J., Ayling R.D., Worth D., Klita M., Steventon A., Line K., Wragg P. (2015). Evaluation of MALDI-ToF as a Method for the Identification of Bacteria in the Veterinary Diagnostic Laboratory. Res. Vet. Sci..

[148] Pereyre S., Tardy F., Renaudin H., Cauvin E., Del Prá Netto Machado L., Tricot A., Benoit F., Treilles M., Bébéar C. (2013). Identification and Subtyping of Clinically Relevant Human and Ruminant Mycoplasmas by Use of Matrix-Assisted Laser Desorption Ionization-Time of Flight Mass Spectrometry. J. Clin. Microbiol..

